# Establishing a Dual Murine Model to Explore the Interactions Between Diabetes and Periodontitis in Mice

**DOI:** 10.3390/ijms26125611

**Published:** 2025-06-11

**Authors:** Bárbara R. Silva, Marco A. R. Hidalgo, Renata C. L. Silva, Erica D. de Avila, Deivys L. P. Fuentes, Iracilda Z. Carlos, Ingrid D. Figueiredo, Estela S. Cerri, Paulo S. Cerri, Amanda M. Baviera, Rafael Scaf de Molon, Raquel M. Scarel-Caminaga

**Affiliations:** 1 Department of Morphology, Genetics, Orthodontics and Pediatric Dentistry, School of Dentistry at Araraquara, São Paulo State University—UNESP, Araraquara 14801-903, SP, Brazil; barbara.roque@unesp.br (B.R.S.); marco.rimachi@unesp.br (M.A.R.H.); renata.cl.silva@unesp.br (R.C.L.S.); estela.sasso@unesp.br (E.S.C.); paulo.cerri@unesp.br (P.S.C.); 2 Department of Diagnosis and Surgery, School of Dentistry at Araçatuba, Sao Paulo State University—UNESP, Araçatuba 16015-050, SP, Brazil; erica.avila@unesp.br; 3 Department of Clinical Analysis, School of Pharmaceutical Sciences, São Paulo State University—UNESP, Araraquara 14800-903, SP, Brazil; deivys.leandro@unesp.br (D.L.P.F.); iracilda.zeppone@unesp.br (I.Z.C.); ingrid.delbone@unesp.br (I.D.F.); amanda.baviera@unesp.br (A.M.B.)

**Keywords:** mouse model, periodontitis, diabetes mellitus, streptozotocin, ligature

## Abstract

This study aimed to develop and validate a dual murine model integrating a high-fat diet (HFD) and a single streptozotocin (STZ) dose to induce diabetes mellitus (DM), alongside periodontitis (Perio) induced by ligature placement and oral inoculation with *Porphyromonas gingivalis* (*P. gingivalis*). The goal was to mimic human pathological conditions, creating a physiologically relevant environment to study the interplay between DM and Perio. A total of 128 six-week-old male C57BL/6J mice were randomly divided into four groups: Control, DM, Perio, and DM-P. DM was induced by HFD and STZ injection, and Perio by ligature placement and *P. gingivalis* infection. Evaluations occurred at baseline and days 7, 14, and 21. Alveolar bone loss was assessed by micro-computed tomography, and inflammation was examined histologically. DM mice showed elevated glucose levels and insulin resistance. Perio and DM-P groups experienced significant bone loss compared with Control and DM groups. The morphometric analysis revealed abundant inflammatory cells and reduced collagen fibers in Perio and DM-P groups, especially at day 7. This dual murine model successfully replicated the key features of DM and Perio, maintaining overall health of the animals, and good tolerability by those subjects to the stress of both interventional procedures.

## 1. Introduction

Oral diseases represent a major global public health concern, affecting approximately 3.5 billion people in 2019, with severe periodontal disease accounting for nearly 1 billion cases [1,2]. Periodontitis is a complex chronic inflammatory condition driven by a dysbiotic shift in the oral microbiota and an exaggerated host immuno-inflammatory response [3]. This disease leads to the destruction of the supporting structures of the periodontium, including the periodontal ligament, cementum, and alveolar bone, and, if left untreated, can ultimately result in tooth loss [3]. Studies conducted from 2011 to 2020 reported a periodontitis prevalence of 61.6%, with moderate to severe forms reaching 53.2% [4]. A variety of systemic conditions influence periodontal health, with diabetes mellitus (DM), particularly type 2 DM (T2DM), playing a significant role in both the development and progression of periodontitis [5,6].

DM is a group of metabolic disorders primarily affecting carbohydrate metabolism, resulting in chronic hyperglycemia [7]. It is also a significant global public health concern, affecting over 500 million adults worldwide in 2021, with projections exceeding 700 million by 2045 [8]. T2DM, the most common form of DM, is characterized by insulin resistance and pancreatic β-cell dysfunction, leading to persistent hyperglycemia. T2DM accounts for approximately 90% of all diabetes cases globally [7,8,9]. A consistently relevant factor in the development of T2DM is its strong association with overweight and obesity. These conditions contribute to the progression of T2DM through complex metabolic mechanisms, including altered adipose tissue function, ectopic fat accumulation in key organs involved in glycemic control, such as the liver, low-grade systemic inflammation, and other related pathways [10,11].

The body of evidence exploring the complex biological interactions between DM, particularly T2DM, and periodontitis continues to grow [12,13]. There is a well-established bidirectional relationship between DM and periodontitis. According to Stöhr et al. [14], patients with DM had a 24% increase in the incidence of developing periodontal disease, and patients with periodontitis had a 26% increase in the incidence of developing DM. A key shared feature is the inflammatory response; systemic inflammation associated with DM can impair periodontal tissue homeostasis, hindering proper tissue repair and regeneration [12]. Conversely, the local inflammation characteristic of periodontitis can contribute to systemic low-grade inflammation [15]. Moreover, virulence factors released by periodontopathogens, such as lipopolysaccharides (LPSs), gingipains, and leukotoxins, can disseminate to critical metabolic organs, including the liver, pancreas, visceral adipose tissue, and skeletal muscle. This may disrupt insulin signaling and contribute to insulin resistance, further complicating glycemic control [13].

Developing effective experimental models that replicate complex diseases such as DM (particularly T2DM) and periodontitis in animals is crucial for advancing our understanding of disease mechanisms and therapeutic strategies. The ligature-induced model is the most widely used and reliable method for inducing periodontitis, consistently resulting in significant alveolar bone loss. The placement of a ligature alone facilitates the accumulation of bacterial plaque around the ligature and causes micro-ulcerations in the sulcular epithelium. This, in turn, increases the permeability of the junctional epithelium and underlying connective tissue to periodontopathogens [16]. The involvement of periodontopathogenic bacteria is particularly important for accurately mimicking human periodontitis. Species such as *Aggregatibacter actinomycetemcomitans* (green complex)*,* and *Porphyromonas gingivalis* (*P. gingivalis*), *Treponema denticola*, and *Tannerella forsythia* (red complex) play central roles in the inflammation-driven dysbiosis that underlies disease progression and tissue destruction [3,17]. While some studies utilize the direct injection of LPSs into the gingival tissue to elicit localized inflammation and simulate aspects of periodontitis [16,18], a more physiologically relevant approach to modeling the microbial challenge involves oral inoculation with *P. gingivalis* in conjunction with ligature placement. This methodology helps establish a more chronic and systemically modulated form of periodontitis. *P. gingivalis* plays a central role in the pathogenesis of periodontitis through virulence factors such as gingipains, LPS, and fimbriae, which drive bacterial adhesion, invasion, tissue destruction, and immune evasion. Beyond its local effects, *P. gingivalis* may also contribute to systemic conditions such as insulin resistance. Emerging evidence indicates that its gingipains can degrade the α-subunit of the insulin receptor in target organs, thereby impairing insulin signaling and supporting a potential link between periodontal inflammation and metabolic dysfunction [19,20].

In the context of DM, the choice of experimental model differs significantly between type 1 (T1DM) and T2DM, reflecting the distinct etiopathogenic mechanisms of each condition. T1DM is an autoimmune disease characterized by the selective destruction of insulin-producing pancreatic β-cells. This process is often associated with the presence of islet-specific autoantibodies and influenced by genetic susceptibility factors. The resulting loss of β-cells leads to insulin deficiency, which in turn causes chronic hyperglycemia and the associated metabolic complications. To study T1DM in experimental settings, streptozotocin (STZ) is commonly used to induce β-cell cytotoxicity. STZ selectively targets pancreatic β-cells, causing their destruction and thereby mimicking the insulin deficiency observed in human T1DM. This model is widely used to investigate the pathophysiology of diabetes, as well as to evaluate potential therapeutic interventions aimed at preserving β-cell function or mitigating the effects of insulin deficiency [7,21]. In contrast, T2DM is strongly associated with obesity, which plays a central role in its development and progression. Excess adipose tissue, particularly visceral fat, contributes to systemic inflammation and metabolic disturbances that lead to insulin resistance. This relationship between obesity and insulin resistance underscores the importance of carefully selecting appropriate animal models that accurately reflect these underlying mechanisms. Models of T2DM often incorporate high-fat diets, genetic predispositions, or a combination of both to induce obesity and mimic the complex metabolic dysfunction seen in human patients. By considering obesity as a key factor, these models enable researchers to study not only glucose dysregulation but also the broader spectrum of metabolic syndrome components, such as dyslipidemia and hypertension, that frequently accompany T2DM. Several genetic models have been developed to study T2DM, including Lep^ob/ob^ mice/Lepr^db/db^ mice—monogenic models of obesity—as well as the Zucker diabetic fatty rats [22,23]. While these models are valuable for investigating obesity-induced insulin resistance and hyperglycemia, they predominantly reflect the genetic component of the disease and do not fully capture the multifactorial nature of T2DM as seen in humans. Therefore, their translational relevance may be limited when it comes to replicating the complex interplay of genetic, environmental, and lifestyle factors involved in human T2DM.

A simpler approach to inducing hyperglycemia in experimental models involves the use of chemical agents such as streptozotocin (STZ) or alloxan. While effective in rapidly elevating blood glucose levels, these models primarily induce pancreatic β-cell damage and do not replicate other critical aspects of T2DM pathogenesis—particularly insulin resistance [21]. As a result, high-fat diet (HFD) models have gained prominence, as they better mimic the natural progression of T2DM. By promoting obesity and metabolic dysfunction, HFD models can induce insulin resistance and impaired glucose tolerance, offering a more physiologically relevant representation of the disease in humans [24].

No single experimental model can fully reproduce all aspects of complex diseases such as DM and periodontitis [25,26]. However, combining different models offers a way to create a more comprehensive and pathophysiologically relevant environment. To date, relatively few studies have utilized such integrative approaches to replicate the phenotypic characteristics of DM and periodontitis when studied together. Given the multifactorial nature of both conditions, in vivo models require careful design to approximate the biological complexity observed in humans. In this study, we aimed to investigate the integration and interaction of an HFD—to increase adipose tissue involvement in metabolism—with a single dose of STZ, used to impair pancreatic function, thereby mimicking key features of T2DM. This metabolic model was combined with two periodontal disease models: the ligature model, which serves as a local irritant and plaque-retentive factor, and oral inoculation with *P. gingivalis*, introducing a microbial challenge. Together, these approaches were designed to closely mimic the pathological conditions observed in humans, thereby providing a complex and physiologically relevant in vivo model to explore the interplay between DM and periodontitis.

## 2. Results

### 2.1. Animal Monitoring

**Figure 1:**
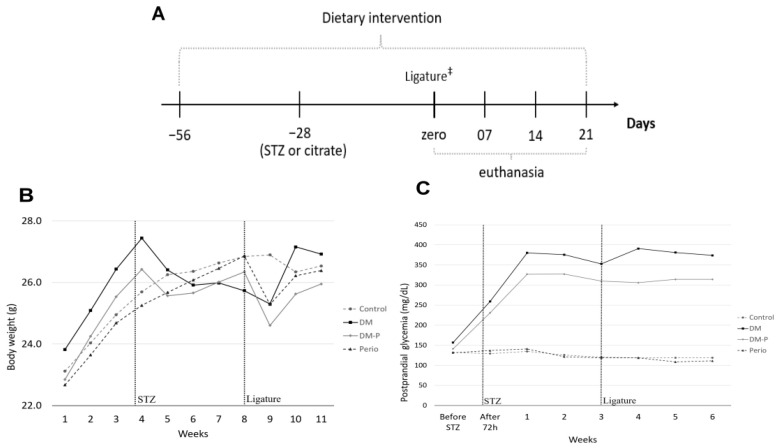
(**A**) Experimental design; ‡ inoculation with live *Porphyromonas gingivalis* in 2% CMC every 2 days for 3 times. (**B**) Body weight (g) of the animals throughout the study period, presented as the weekly mean. (**C**) Postprandial blood glucose (mg/dL) of the animals throughout the study period (46 days), presented as the weekly mean. STZ—streptozotocin.

To monitor the gain and/or loss of body weight in the animals, we conducted weekly weighing of C57BL/6J mice. Mice fed with HFD (DM and DM-P groups) exhibited an increase in body weight compared with animals on a standardized mouse control diet (Perio and Control groups). However, following the STZ injection, the HFD-fed mice experienced a weight loss, as depicted in Figure 1B and in Table 1. A further slight decrease in body weight was observed in the groups that underwent ligature placement (DM-P and Perio groups). This was an expected reaction, since it would be difficult for the animals to feed in the first week with the ligature around the tooth. Despite these fluctuations in body weight due to the surgical procedures, anesthesia, and treatments employed, stabilization of body weight occurred in the subsequent weeks, without statistical significance among groups.

To further elucidate the effectiveness of our DM-mouse model, postprandial blood glucose levels (Figure 1C) clearly demonstrated higher glucose levels in the mice subjected to experimental diabetes induction (DM and DM-P groups) compared with the non-diabetic groups (Perio and Control). This confirmed the persistence of hyperglycemia in the DM and DM-P groups throughout the experimental period.

### 2.2. Lipid and Carbohydrate Marker Levels

Supporting the results of hyperglycemia in the animals, and in line with the postprandial blood glucose findings, we found that fasting blood glucose levels were consistently higher in the DM group than in the Control group (baseline: *p* < 0.0001; 7 days: *p* = 0.0006; 14 days: *p* < 0.0001; 21 days: *p* = 0.0096) and the Perio group (7 days: *p* = 0.0008; 14 days: *p* < 0.0001; 21 days: *p* = 0.0104). Similarly, the DM-P group showed higher levels than both the Control (7 days: *p* < 0.0001; 14 days: *p* = 0.0035; 21 days: *p* = 0.0103) and Perio groups (7 days: *p* < 0.0001; 14 days: *p* = 0.0059; 21 days: *p* = 0.0113), as shown in Table 1. No significant differences in glucose levels were observed between the DM and DM-P groups (7 days: *p* = 0.5408; 14 days: *p* = 0.4033; 21 days: *p* > 0.9999) or between the Perio and Control groups (7 days: *p* = 0.9753; 14 days: *p* = 0.9964; 21 days: *p* = 0.8448). Additionally, no significant differences in plasma insulin levels were detected among the groups.

The DM group exhibited higher levels of HOMA-IR than the Control group (baseline: *p* < 0.0001; 7 days: *p* = 0.0106; 14 days: *p* = 0.0007; 21 days: *p* = 0.0381) and the Perio group (7 days: *p*= 0.0221; 14 days: *p* = 0.0013; 21 days: *p* = 0.0440). Similarly, the DM-P group showed higher levels of HOMA-IR than both the Control (7 days: *p* < 0.0001; 14 days: *p* = 0.0231; 21 days: *p* = 0.0253) and Perio groups (7 days: *p* < 0.0001; 14 days: *p* = 0.0390; 21 days: *p* = 0.0262), indicating insulin resistance in the diabetic groups.

The HFD-fed groups showed elevated total cholesterol levels at baseline (DM group: *p* = 0.0006) and at day 7 (DM group: *p* = 0.0346 and DM-P group: *p* = 0.0209) compared with the Control group (Table 1). By day 14, only the DM group showed significantly higher cholesterol levels than the Control group (*p* = 0.0275). Similarly, HDL cholesterol levels were higher in the DM group at both baseline (*p* = 0.0003) and day 14 (*p* = 0.0321) than in the Control group. A similar trend was observed in the DM-P group; however, the difference was not statistically significant. No significant differences in triglyceride levels were observed among the groups.

### 2.3. Alveolar Bone Loss

**Figure 2:**
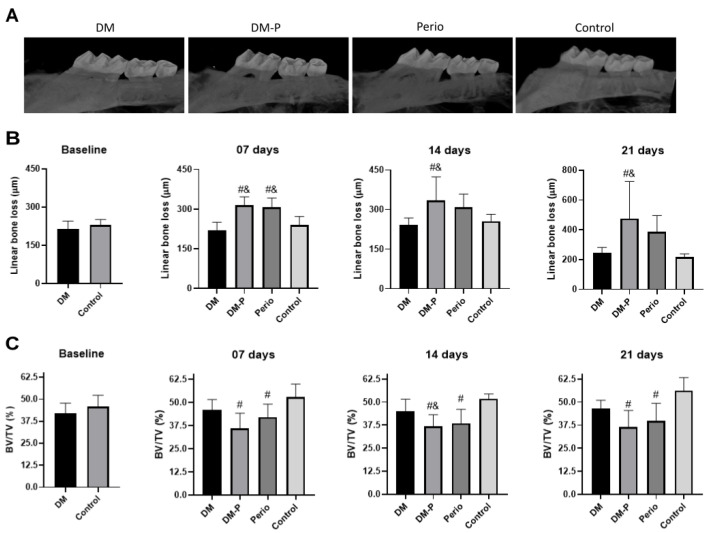
(**A**) Images obtained from the three-dimensional reconstruction by micro-CT of the maxillary molars of each experimental group at 21 days. (**B**) Mean and standard deviation (SD) of the linear bone loss (µm) in the interproximal region between the 1st and 2nd maxillary molars on the right side of all groups, at each experimental time point. (**C**) Mean and SD of the percentage of bone volume obtained from the region of the 1st to 2nd maxillary molars on the right side of all groups at each experimental time point. # Significant difference compared with the Control group; & significant difference compared with the DM group.

To verify the effectiveness of oral inoculation with *P. gingivalis* associated with ligature placement to induce bone loss, we performed micro-CT analysis. As expected, the Control group demonstrated the lowest linear bone distance (CEJ to ABC distance), which denotes bone preservation (Figure 2B). Animals of the DM group showed similar CEJ to ABC distance to the Control group (baseline: *p* = 0.1562; 7 days: *p* = 0.7017; 14 days: *p* = 0.9421; 21 days: *p* = 0.9876), which denotes that experimental DM did not enhance bone destruction in mouse. By contrast, the DM-P group showed greater linear bone distance than the DM group (7 days: *p* < 0.0001; 14 days: *p* = 0.0090; 21 days: *p* = 0.0253) and the Control group (7 days: *p* = 0.0010; 14 days: *p* = 0.0346; 21 days: *p* = 0.0222) (Figure 2B). At 7 days post-ligature placement, the Perio group demonstrated the greatest linear bone distance compared with the DM (*p* = 0.0003) and Control groups (*p* = 0.0032). Interestingly, combining periodontitis with diabetes mellitus did not worsen periodontal breakdown in the conditions studied.

In terms of alveolar bone volume loss (BV/TV%) (Figure 2C), and similar to the findings from the linear measurements, both groups subjected to experimental periodontitis induction exhibited significant lower bone volume in the region between the first and second maxillary molars than in the Control group (Perio group: 7 days: *p* = 0.0285, 14 days: *p* = 0.0008, 21 days: *p* = 0.0045; DM-P group: 7 days: *p* = 0.0006, 14 days: *p* = 0.0002, 21 days: *p* = 0.0013). While the DM-P group demonstrated greater tissue impairment, no significant differences were observed compared with the Perio group (7 days: *p* = 0.3206; 14 days: *p* = 0.9401; 21 days: *p* = 0.8366). Interestingly, the DM group showed a slight decrease in BV/TV compared with the control group throughout the evaluation period, but no significant differences between the groups were observed (baseline: *p* = 0.0714; 7 days: *p* = 0.3138; 14 days: *p* = 0.1506; 21 days: *p* = 0.2138).

### 2.4. Inflammatory Process of Gingival Tissues

To complement the findings observed by micro-CT, we performed morphometric analysis of the gingival tissue. We emphasize that only the ‘gingival collar’ around the right first molar was examined. This selection was based on the disease induction model, the specimen size, and the need to maximize the available tissue for complementary analyses beyond micro-CT.

**Figure 3:**
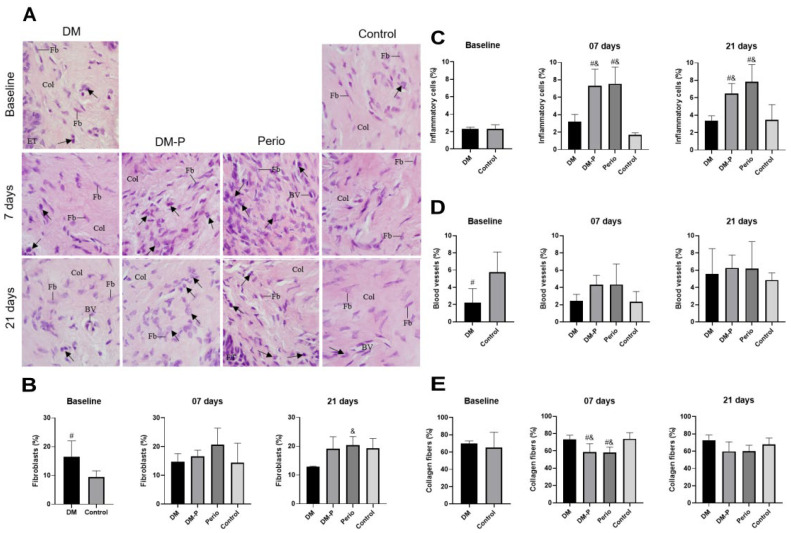
(**A**) Light micrographs of portions of gingival mucosa showing the lamina of mice. Arrow—inflammatory cell; Fb—fibroblasts; BV—blood vessel; ET—epithelial tissue; Col—collagen fibers. Scale Bars: 35 µm. (**B**–**E**) Graphics illustrating the volume density of fibroblasts (**B**), inflammatory cells (**C**), and blood vessels (**D**) and volume density of collagen fibers in the lamina propria of gingival mucosa of mice from DM, DM-P, Perio, and Control groups. # Significant difference compared with the Control group; & significant difference compared with the DM group. Values expressed as mean and standard deviation.

After the induction of periodontitis, several inflammatory cells were observed between collagen fibers in the DM-P and Perio groups, particularly at 7 days post-disease induction (Figure 3A). Although no significant difference was found in the frequency of fibroblasts among groups (Figure 3B), a significant increase in the frequency of inflammatory cells was observed in the DM-P and Perio groups in comparison with DM (DM-P group: 7 days: *p* = 0.0040 and 21 days: *p* = 0.0173; Perio group: 7 days: *p* = 0.0017 and 21 days: *p* = 0.0010) and Control groups (DM-P group: 7 days: *p* = 0.0009 and 21 days: *p* = 0.0307; Perio group: 7 days: *p* = 0.0004 and 21 days: *p* = 0.0021) (Figure 3C). At 7 days, the content of collagen was significantly lower in the DM-P and Perio than in DM (DM-P group: *p* = 0.0218; Perio group: *p* = 0.0139) and Control groups (DM-P group: *p* = 0.0402; Perio group: *p* = 0.0292), but no significant difference in the collagen content was detected among the groups at 21 days (Figure 3E).

## 3. Discussion

The complex interplay between periodontitis and DM is rooted in a bidirectional relationship, where each condition influences the onset, progression, and severity of the other [27,28,29]. T2DM, is characterized by chronic hyperglycemia and insulin resistance, which impair immune function and wound healing. These alterations compromise the host’s ability to control oral microbial biofilms, contributing to the initiation and progression of periodontitis [30]. Conversely, periodontitis, a chronic inflammatory disease affecting the supporting structures of the teeth, can exacerbate systemic inflammation through the release of pro-inflammatory cytokines (e.g., tumor necrosis factor alpha and interleukin (IL)-1 and IL-6), bacterial endotoxins, and oxidative stress [6]. These systemic inflammatory mediators may worsen insulin resistance and glycemic control, fueling a vicious cycle of inflammation and metabolic dysfunction. Understanding this interplay is essential for public health due to the high prevalence and global burden of both diseases. However, unraveling the causal mechanisms and temporal dynamics of their interaction in humans is challenging due to ethical and practical limitations. Animal models that combine experimental T2DM and periodontitis are thus crucial tools for dissecting the mechanistic links between these diseases [31,32]. They allow controlled manipulation of variables such as diet, microbial challenge, genetic background, and immune response. By mimicking the human pathophysiology in a controlled setting, these models can (1) clarify how hyperglycemia and insulin resistance affect periodontal inflammation and bone resorption; (2) elucidate how periodontal pathogens contribute to systemic metabolic disturbances; (3) identify molecular pathways and immune responses shared or altered in comorbidity; (4) provide a platform to test novel therapeutic strategies targeting one or both diseases simultaneously (e.g., anti-inflammatory agents, hydrogel with antimicrobials, immunomodulators, or metabolic regulators) [33,34,35]. Therefore, combined animal models of DM and periodontal inflammation are indispensable for advancing our understanding of this bidirectional relationship and for developing more effective, targeted, and personalized treatment approaches.

In this study, we employed a dual experimental model in mice to enable the development of complex biological interactions between DM and periodontitis, closely replicating the conditions observed in humans. The effectiveness of disease induction was confirmed for DM, evidenced by fasting blood glucose levels exceeding 200 mg/dL and increased HOMA-IR values, and for experimental periodontitis, as demonstrated by volumetric bone loss and increased volume density of inflammatory cells. Notably, the simultaneous induction of both conditions in the same group of animals was well tolerated. The main advantage of this combined murine model lies in its ability to create a biologically realistic environment that includes both lipid metabolism dysfunction and periodontal colonization by pathogens commonly found in severely affected patients. This approach enables a more accurate and controlled investigation of the interrelationship between these two complex diseases and facilitates their manipulation in an experimental setting.

To model T2DM, we used a high-fat diet (HFD; 35% lipids) in combination with a single dose of STZ, successfully reproducing key features of T2DM pathogenesis, such as, insulin resistance and hyperglycemia [24]. Here, male C57BL/6J mice were purposefully selected due to their well-documented sensitivity to HFD and ability to develop a chronic metabolic response when maintained on the prescribed diet over time [36,37]. The 8-week duration of diabetes induction was chosen to allow the establishment of these chronic metabolic changes. Although STZ is commonly used to induce T1DM, primarily by causing direct pancreatic β-cell damage, it does not by itself reproduce the multifactorial etiology of T2DM in humans [21]. In contrast, T2DM in dyslipidemic or obese patients is characterized by chronic hyperglycemia, insulin resistance, and disrupted lipid metabolism. In mice, chronic HFD consumption induces morphological and functional alterations in adipose tissues, including increased visceral adiposity, secretion of pro-inflammatory adipocytokines, and ectopic fat accumulation in non-adipose tissues, changes that contribute to lipotoxicity and metabolic dysfunction [36]. To contribute to these pathological alterations, corn starch and dextrinized corn starch were included in the composition of the HFD used in this study. According to Winzell and Ahrén (2004) [38], these components play a critical role in the development of fasting hyperglycemia and insulin resistance. Furthermore, the HFD composition triggers multiple inflammatory pathways, as evidenced by significant increases in pro-inflammatory cytokines such as TNF-α and IL-6 in the liver and kidneys of animals fed this diet. These inflammatory responses contribute to systemic metabolic dysfunction and are consistent with findings from previous studies using the same HFD formulation as in the present investigation [39].

The HFD was maintained throughout the entire 11-week experimental period. During the first four weeks, we observed an increase in body weight in the animals. Following STZ administration, body weight decreased and subsequently stabilized. Similar findings were reported by Wang et al. [40] who observed reduced body weight in C57BL/6J mice after administering multiple low doses of STZ (55 mg/kg for 5 days), compared with both healthy controls and genetic models such as db/db and TallyHo/JngJ mice. Likewise, in a study by Huang et al. [41] rats fed an HFD and given a low dose of STZ (35 mg/kg) also exhibited a decline in body weight following STZ injection.

When analyzing HDL and total cholesterol levels, both were similarly elevated in the DM and DM-P groups (Table 1). In mice, HDL is the predominant lipoprotein involved in the transport of cholesterol in plasma, which largely accounts for the parallel increase observed between HDL and total cholesterol levels. This characteristic differs from humans, where low-density lipoprotein (LDL) plays a more prominent role in cholesterol transport. Consequently, changes in total cholesterol in mice are often driven by alterations in HDL levels. Although a bidirectional relationship between HDL dysfunction and T2DM has been well-documented in humans [42], the physiological significance and functional role of HDL in murine models of diabetes remain less well understood. Further research is therefore warranted to determine the optimal range and function of HDL in mice, especially in the context of metabolic disorders such as T2DM, to better align findings from murine studies with human pathophysiology.

In our study, a single STZ dose of 120 mg/kg successfully accelerated the onset of hyperglycemia, which was evident 72 h after administration and remained above 200 mg/dL for over 46 days. This approach was well tolerated by the animals. Hahn et al. [43] compared the effects of a single high-dose (150–200 mg/kg) versus multiple low-dose (50 mg/kg for 5 days) STZ regimens in mice and found that a substantial proportion of pancreatic β cells remained functional regardless of the dosing strategy. Their study reported increased insulin staining intensity and downregulation of GLUT-2 expression in the pancreas across both regimens, with no significant differences between doses. These results suggest that, while the high-dose model triggers hyperglycemia more rapidly, the low-dose regimen leads to a more gradual β cell loss. Nonetheless, both approaches ultimately exert a similar detrimental effect on pancreatic function by the third week. Taking into account the need for multiple interventions (DM and periodontitis induction) and aiming to reduce animal handling and stress, we opted for the single STZ dose in our model. This choice balanced effective diabetes induction with good animal tolerance.

Elevated fasting blood glucose and insulin resistance, measured using HOMA-IR, are key indicators of successful T2DM modeling. In our study, fasting glucose levels were significantly higher in the DM and DM-P groups compared with the Perio and Control groups throughout the experimental period. Notably, insulin levels remained comparable between HFD/STZ-treated mice and those fed a standard diet across all time points. This finding suggests that pancreatic damage caused by the single STZ dose was not extensive enough to result in hypoinsulinemia. Such a result is highly desirable in T2DM animal models, as early stages of human T2DM are typically marked by insulin resistance rather than insulin deficiency. Our findings are consistent with those of Hahn et al. [43], who reported increased insulin staining intensity in pancreatic β cells two weeks after STZ administration, regardless of whether a single high dose or multiple low doses were used.

Regarding insulin resistance, primarily assessed by HOMA-IR, our study, using the HFD/STZ model adapted from Li et al. [44], demonstrated significantly higher HOMA-IR values in the DM and DM-P groups compared with the normoglycemic groups throughout the experimental period. These findings indicate that our murine DM model successfully reproduced insulin resistance alongside hyperglycemia. Replicating this phenotype is particularly valuable for preclinical investigations of T2DM, as insulin resistance is a hallmark feature in human T2DM, leading to hyperglycemia and a progressive decline in pancreatic function [9]. Chao et al. [45] compared insulin resistance across four non-genetic T2DM induction models in rats—fructose-rich diet, HFD/STZ, low-dose STZ, and nicotinamide + STZ injection—and concluded that the fructose-rich and HFD/STZ models were most effective at elevating HOMA-IR and showed the lowest responsiveness in the hyperinsulinemic euglycemic clamp test (the gold standard for assessing insulin sensitivity), confirming their ability to induce insulin resistance. Similarly, Racine et al. [37], in a study evaluating sex-specific responses to a 60% kcal fat diet combined with multiple low-dose STZ injections (30 mg/kg for 3 days), reported fasting hyperinsulinemia and increased HOMA-IR in male C57BL/6J mice.

Insulin resistance has also been linked to virulence factors from periodontal pathogens and the presence of chronic low-grade inflammation [13,46]. In this study, we aimed to replicate periodontitis as closely as possible to the human condition. To this end, we employed a combined model using ligature and *P. gingivalis* oral inoculation, incorporating a pathogen typically associated with subgingival microbial dysbiosis, especially under systemic inflammatory conditions [3,47]. The ligature model alone effectively induces rapid alveolar bone loss, which typically elicits an acute local inflammatory response that peaks within two weeks, and thereafter is subsequently stabilized [18,25]. In contrast, *P. gingivalis* gavage requires a longer stimulation period—exceeding two weeks—and a prolonged follow-up to assess alveolar bone loss, with effects that persist even after the stimulus is removed [47]. Combining these two approaches, therefore, offers advantages for both short- and long-term evaluations and strengthens our model’s translational relevance. Additionally, the C57BL/6J mouse strain was chosen due to its established use in modeling both diabetes and periodontitis. Although this strain is not the most susceptible to *P. gingivalis* oral inoculation-induced periodontitis [16,26], we selected the highly virulent *P. gingivalis* W83 strain for our experiments. This strain is frequently isolated in moderate to severe periodontitis and is known for its high pathogenic potential, particularly due to its active gingipains [48,49].

Using the combined ligature and *P. gingivalis* oral inoculation model, we observed alveolar bone loss and an increased volume density of inflammatory cells in the gingival tissue of the DM-P and Perio groups, particularly at day 7, with persistence of these features throughout the 21-day experimental period. Our findings align with those of de Molon et al. [25], who reported that the ligature model was most effective in inducing gingival inflammation and bone loss in C57BL/6 mice between days 7 and 15, compared with mono/polymicrobial gavage or local injection of heat-killed *P. gingivalis*. Similarly, Palioto et al. [47] demonstrated that *P. gingivalis* gavage (administered six times on alternate days) effectively induced bone loss even when combined with ligature placement, despite the ligature being removed five days after placement. In our study, despite administering *P. gingivalis*, we did not observe an exacerbation of alveolar bone loss or gingival inflammation at 21 days. This may be attributed to the relatively short colonization period, as only three gavage applications were performed, despite the use of a highly pathogenic bacterial strain.

According to a review by Dal Acqua et al. [18], periodontitis induction models involving microbial challenges with periodontopathogens are likely more suitable for evaluating systemic effects due to their chronic inflammatory nature. Supporting this, Blasco-Baque et al. [50] assessed the impact of adaptive immune responses on metabolic disturbances using a four-week microbial challenge with *P. gingivalis*, *Fusobacterium nucleatum* (Fn), and *Prevotella intermedia* (Pi), followed by HFD feeding for three months. They observed increased systemic adaptive immune activation, and animals with greater glucose intolerance exhibited reduced serum anti-*P. gingivalis* antibody levels. Nonetheless, in the present study, we selected *P. gingivalis* W83 as a mono-inoculum due to its well-documented virulence and established role as a keystone pathogen in the pathogenesis of periodontitis [16]. The W83 strain, in particular, is recognized for its ability to disrupt host-microbial homeostasis, subvert immune responses, and initiate alveolar bone loss even in the absence of other bacterial species [48,49].

The negative impact of experimental periodontitis on glucose metabolism—such as increased glucose levels in oral glucose tolerance tests and higher HOMA-IR values—has been demonstrated in several models, including combined ligature + *P. gingivalis* [51], polymicrobial colonization [50], and ligature alone [52]. In our study, we did not observe significant differences in fasting blood glucose levels or HOMA-IR when comparing the DM-P group with the DM group, or the Perio group with the Control group. The absence of these systemic alterations may be attributed to the limited duration of *P. gingivalis* exposure, which was shorter than in previous studies [47,50,51]. Moreover, the total duration of periodontitis evaluation in our study—21 days from the onset of stimulation—was also shorter than the periods adopted in those studies [47,50,51]. Nevertheless, this timeframe was sufficient to elicit key morphological features of periodontitis, including alveolar bone loss and increased infiltration of inflammatory cells in the gingival tissue. These findings suggest that both *P. gingivalis* stimulation protocol and the relatively short follow-up period may have contributed to the lack of observable systemic effects in our results.

The exacerbation of alveolar bone loss in hyperglycemic animals with experimental periodontitis, compared with normoglycemic animals, has been reported in several studies [51,52,53]. Consistent with these findings, the DM-P group in our study exhibited the most severe linear bone loss and the lowest BV/TV ratio. Although these values were not statistically different from those observed in the Perio group (normoglycemic), they suggest a detrimental effect of hyperglycemia on periodontitis progression. The impairment of local periodontal responses in hyperglycemic conditions was also demonstrated by Xiao et al. [54], who reported elevated Il-17a gene expression in the gingiva of db/db mice even in the absence of experimentally induced periodontitis. Their study also found increased IL-6 and RANKL-positive cells, spontaneous alveolar bone loss, and changes in the oral microbiota of hyperglycemic mice compared with normoglycemic controls. Similarly, Zhang et al. [53] showed that M1 macrophage polarization was enhanced in high-glucose environments under LPS stimulation compared with low-glucose conditions. Furthermore, M1 macrophages differentiated in a high-glucose/LPS setting significantly promoted osteoclast formation in vitro.

This study has some limitations, primarily due to our concern about overburdening the animals with multiple and prolonged procedures. We used *P. gingivalis* W83 as a mono-inoculum for a relatively short period, and the follow-up duration of 21 days was also limited. Future studies should explore longer periods of *P. gingivalis* colonization and consider using multi-species inocula to more accurately mimic chronic oral infection. Additionally, extending the observation period would allow better assessment of long-term systemic effects, such as insulin resistance. Despite these limitations, our study contributes valuable insights to the complex and clinically significant relationship between periodontitis and diabetes, emphasizing the importance of further research for global public health. Preclinical studies employing experimental models that closely resemble the human condition are essential to deepen our understanding of this bidirectional interaction. This study advances the development of a robust preclinical combined model suitable for investigating the interplay between diabetes and periodontitis in mice. Finally, two key insights emerged from this study: (i) the use of a single high-dose STZ protocol effectively accelerated diabetes onset without markedly altering insulin or lipid levels, yielding results comparable to those of multiple low-dose protocols; (ii) the combined model was well tolerated by the animals, even in those exposed to the stress of both metabolic and inflammatory interventions.

## 4. Materials and Methods

### 4.1. Animals and Experimental Design

One hundred and twenty-eight 6-week-old male C57BL/6J mice, weighing approximately 25 g, were housed in polypropylene cages (four mice per cage) under controlled conditions, with a temperature of 25 °C (±2 °C), controlled humidity, and a 12-h light/dark cycle. Food and water were provided ad libitum at the animal facilities of the School of Dentistry at Araraquara (UNESP). This project was approved by the Ethics Committee on Animal Experimentation of UNESP (CEUA-UNESP 26/2020), and adhered to the ethical standards outlined in the Guide for the Care and Use of Laboratory Animals [55].

Mice were randomly allocated to four experimental groups (n = 8 per group per time point): the DM group, in which diabetes was induced without periodontitis; the DM-P group, in which both diabetes and periodontitis were induced; the Perio group, periodontitis was induced without DM; and the Control group, no DM or periodontitis was induced.

### 4.2. Experimental DM Model

The experimental diabetes model was established using an HFD combined with STZ administration. After a 2-week acclimatization period, mice in the DM and DM-P groups were fed an HFD (5.40 kcal/g, 35% lipids; PragSoluções Biociências, Domeneghetti e Corrêa Ltd.a, Jaú, SP, Brazil) (Table 2) for 4 weeks [44]. Subsequently, STZ (120 mg/kg), prepared in 0.1 M sodium citrate buffer (pH 4.5), was administered intraperitoneally. The HFD was provided for an additional 4 weeks, totaling 8 weeks prior to the induction of periodontitis, and was maintained throughout the remainder of this study. Body weight was monitored weekly for each mouse. This study’s experimental design is outlined in Figure 1A.

Blood glucose levels were measured one week prior to STZ administration, 72 h after administration, and weekly thereafter until this study’s conclusion. Measurements were taken in the morning following a two-hour fasting period (postprandial) using a portable digital glucometer (Abbott Diabetes Care Ltd.a, São Paulo, SP, Brazil), with blood samples collected from the tail.

The Perio and Control groups were fed a control diet (3.85 kcal/g, 4% lipids; PragSoluções Biociências, Domeneghetti e Corrêa Ltd.a, Jaú, SP, Brazil) [56] for 4 weeks. Instead of STZ, these groups received intraperitoneal administration of 0.1 M sodium citrate buffer (pH 4.5) as a vehicle injection control to determine whether the vehicle itself had any effects. This control diet was maintained throughout the study period.

### 4.3. Experimental Periodontitis Model

It is worth mentioning that animals euthanized at baseline did not undergo periodontitis induction and were only assigned to the DM and Control groups during this period. In the subsequent time points—7, 14, and 21 days—all four groups (DM, DM-P, Perio, and Control) were evaluated. Experimental periodontitis in the DM-P and Perio groups was induced using a combination of two methods: ligature placement and colonization by *P. gingivalis*.

#### 4.3.1. Cultivation of *P. gingivalis* 

*P. gingivalis* W83 was cultured on blood agar medium supplemented with 1 μL/mL menadione and 5 μL/mL hemin to meet its nutritional requirements. The cultures were maintained in an anaerobic jar containing 90% N_2_ and 10% CO_2_ in an incubator at 37 °C for 10 days. A growth curve was established to determine the exponential phase, measuring the optical density (OD) at 600 nm and estimating the concentration of microorganisms in CFU/mL (colony-forming units) [57]. After 48 h, colonies were transferred from the plate to a 15 mL tube of BHI broth supplemented with hemin and menadione, and maintained anaerobically for another 48 h. The OD of the 48-h inoculum was adjusted to 1.4 for subsequent dilution in 10 mL of supplemented BHI broth. On the day of bacterial inoculation, the bacterial culture was mixed with 2% carboxymethylcellulose (CMC) to reach a final concentration of 1 × 10^9^ CFU. Therefore, an OD 600 of approximately 1.4 corresponded to ~1 × 10^9^ CFU/mL. In sequence, a volume of 100 μL of this mixture was applied directly onto the ligatures in the oral cavities of the animals undergoing ligature placement using a micropipette with a sterile tip [25].

#### 4.3.2. Ligature Placement

This procedure was adapted from previous studies with slight modifications [25,26,58]. Mice in the DM-P and Perio groups were anesthetized with a combination of ketamine hydrochloride (0.08 mL/100 g body weight) and xylazine hydrochloride (0.04 mL/100 g body weight). The animals were positioned on a specialized operating table designed to keep their mouths open, allowing access to the posterior maxillary teeth.

Ligatures were placed by subgingivally threading a 6-0 nylon suture around the first maxillary molars on both sides. After ligature placement, 100 μL of the *P. gingivalis* mixture in 2% CMC was topically applied onto the ligatures in the oral cavity using a micropipette with a sterile tip. The animals were deprived of food and water for the next two hours following the application. Two days later, a fresh preparation of the *P. gingivalis* mixture in 2% CMC was applied in the same manner, followed by another two-hour food and water deprivation. This process was repeated once more after another two days, resulting in a total of three applications.

### 4.4. Analysis of Lipid and Carbohydrate Metabolism Markers

The animals were euthanized at baseline, and at 7, 14, and 21 days following the experimental induction of periodontitis. Prior to euthanasia, the animals were fasted for 12 h and anesthetized via intraperitoneal injection of xylazine (16 mg/kg) and ketamine (90 mg/kg). Once fully anesthetized, euthanasia was carried out by total exsanguination via cardiac puncture. Whole blood was collected from each animal and immediately transferred into tubes containing EDTA/K3 as an anticoagulant.

Plasma was promptly separated from each blood sample using a Histopaque gradient (1119 and 1077, Sigma-Aldrich, St. Louis, MO, USA). To prepare the gradient, 0.5 mL of Histopaque 1119 was added to a 15 mL conical tube, followed by 0.5 mL of Histopaque 1077. Subsequently, 1 mL of whole blood was carefully layered on top. Separation was achieved via centrifugation at 400× *g* for 35 min at room temperature (25 °C) with minimal acceleration (a = 4), no deceleration (b = 0), and no brake. The plasma obtained was aliquoted and stored in an ultrafreezer.

A plasma aliquot from each animal was analyzed at a Clinical Analysis Laboratory using the AU480 Chemistry Analyzer (Beckman Coulter, Brea, CA, USA). Beckman Coulter reagents were used to measure glucose, triglycerides, and cholesterol using different enzymatic methods; glucose was measured using the glucose oxidase method, triglycerides with lipase and glycerokinase, and total cholesterol with cholesterol esterase and cholesterol oxidase. Plasma insulin levels were measured using the ELISA (enzyme-linked immunosorbent assay) technique with the mouse INS (insulin) ELISA kit (E-EL-M1382, Elabscience, Houston, TX, USA), according to the manufacturer’s instructions. Samples were diluted 1:2 with the kit’s sample diluent, and the homeostasis model assessment of insulin resistance (HOMA-IR) was calculated according to the formula [59]:

HOMA-IR = [fasting glucose (mmol/L) × fasting insulin (μU/mL)]/22.5

### 4.5. Microcomputed Tomography Analysis (Micro-CT)

Dissected hemimaxillae (n = 8 per group) were carefully collected, fixed in 4% formaldehyde (prepared from paraformaldehyde) for 48 h, and then stored in 70% ethanol prior to micro-CT scanning. The right hemimaxillae were scanned using a high-resolution micro-CT scanner (Skyscan 1272; Bruker, Billerica, MA, USA) at the School of Dentistry at Araçatuba (FOA) UNESP.

The scanning parameters were established based on previous studies by our research group [25,26,60], according to the following settings: X-ray beam tube voltage was set at 70 kV, pixel size was 8.75 µm, and a 0.5 mm aluminum filter was used. Linear analysis was performed by accurately aligning the reconstructed images (NRecon software, version 1.6.10.2, Bruker, Billerica, MA, USA) from the sagittal section using DataViewer software (version 1.5.1.2, Bruker, Billerica, MA, USA). The distance (in µm) from the cemento-enamel junction (CEJ) to the alveolar bone crest (ABC) was measured on the distal surface of the distopalatal root of the maxillary first molar and the mesial face of the mesiopalatal root of the second molar.

In CTan software (CTAnalyzer, version 1.15.4.0, Bruker, Billerica, MA, USA), a region of interest (ROI) was delineated from the root apices to the alveolar crest, and from the mesial root of the first molar to the distal root of the second molar using axial images for analysis of volumetric bone alterations. Dental roots and the periodontal ligament were excluded from the ROI. The ROI consisted of 50 slices extending from the CEJ to the apical portion of the root. Bone tissue was manually outlined at regular intervals using a slice-based method every 10 planes with an interpolation tool. The roots were then manually outlined in the 50 selected slices and excluded from the bone analysis. As a result, the entire bone area, excluding roots, was included in the ROI, and bone volume (BV), tissue volume (TV), and bone volume fraction (BV/TV, %) were assessed.

### 4.6. Morphometric Analysis of Gingival Tissue

The gingival tissue around the first maxillary molar on the right side of the animal, the same site analyzed by micro-CT, was collected and immediately immersed in 4% formaldehyde (prepared from paraformaldehyde) buffered at pH 7.2 with 0.1 M sodium phosphate for 48 h at room temperature. After fixation, the gingival samples were dehydrated, clarified in xylene, and embedded in paraffin. The histological sections were stained with hematoxylin and eosin (H&E), and two non-serial images of the connective tissue (lamina propria) per animal (n = 6 per group) were captured at 100× magnification using a camera (DP71, Olympus, Tokyo, Japan) attached to a light microscope (BX51, Olympus, Tokyo, Japan).

A grid system containing 234 points was constructed using an image analysis system (Image-Pro Plus 6.0 software, Media Cybernetics, Rockville, MD, USA) and overlaid on digital images on each image of lamina propria. The number of intersections (points) over the following components was quantified: inflammatory cells, blood vessels, fibroblasts, collagen fibers, extracellular spaces, and other structures (epithelial tissue, spaces). The inflammatory cells were identified based on their morphological features, such as multilobed nucleus (neutrophils), rounded cell with round and dense nucleus (lymphocytes), ovoid/elliptical cells with eccentrically located nucleus (plasma cells), and irregularly shaped cells with indented nucleus (macrophages), while fibroblasts showed an elliptical/flattened nucleus shape [61,62]. Considering that 234 points represented 100%, the percentage value (volume density) of each component was calculated for each animal from each group [26,61,63,64].

### 4.7. Statistical Analyses

Graphs monitoring body weight and postprandial blood glucose levels were generated using Microsoft Excel 2024. Data normality was assessed using the Shapiro–Wilk test. For multiple group comparisons, one-way ANOVA followed by Tukey’s post hoc test was employed, while comparisons between two groups were conducted using a *t*-test. All statistical analyses were performed using GraphPad Prism software (version 8.4.3), with statistical significance defined as a *p*-value ≤ 0.05.

## 5. Conclusions

The experimental models for diabetes and periodontitis effectively reproduced the phenotypes of each disease, both individually and in combination, with good tolerance observed in animals subjected to the combined protocols. Our findings make a valuable contribution to preclinical research on periodontitis–diabetes comorbidities by successfully integrating two complex disease models within the same group of animals. This approach enabled a more realistic representation of each disease’s pathophysiology and their interactions, enhancing the translational relevance of our study.

## Figures and Tables

**Table 1:** Body weight (g) and plasmatic levels of markers related to lipid and carbohydrate metabolism of each group during the experimental time points.

	Time Point	DM	DM-P	Perio	Control
**Physical parameter**					
Body weight (g)	baseline	23.5 (±2.9) ^b^	-	-	27.5 (±1.2) ^a^
	7 days	27.0 (±2.1) ^a^	25.6 (±1.6) ^a^	25.2 (±2.6) ^a^	25.2 (±1.7) ^a^
	14 days	27.9 (±2.4) ^a^	25.4 (±2.1) ^a^	26.0 (±2.0) ^a^	26.4 (±2.2) ^a^
	21 days	26.0 (±1.8) ^a^	27.0 (±1.8) ^a^	27.1 (±2.0) ^a^	26.7 (±1.7) ^a^
**Plasma markers**					
Fasting blood glucose (mg/dL)	baseline	278.5 (±57.8) ^a^	-	-	92.7 (±31.2) ^b^
	7 days	238.0 (±99.4) ^a^	284.3 (±74.6) ^a^	91.1 (±24.0) ^b^	78.9 (±27.8) ^b^
	14 days	281.1 (±92.1) ^a^	228.7 (±81.1) ^a^	111.8 (±28.2) ^b^	105.1 (±21.9) ^b^
	21 days	235.3 (±106.7) ^a^	233.8 (±68.7) ^a^	98.7 (±23.0) ^b^	60.0 (±20.0) ^b^
Insulin (pg/mL)	baseline	75.4 (±11.1) ^a^	-	-	84.2 (±15.9) ^a^
	7 days	80.5 (±7.9) ^a^	92.9 (±10.4) ^a^	89.0 (±12.8) ^a^	90.3 (±9.6) ^a^
	14 days	98.7 (±14.8) ^a^	96.0 (±15.9) ^a^	97.2 (±9.1) ^a^	94.3 (±18.1) ^a^
	21 days	99.9 (±14.4) ^a^	111.0 (±13.1) ^a^	103.9 (±19.6) ^a^	99.2 (±37.3) ^a^
Triglycerides (mg/dL)	baseline	33.7 (±9.4) ^a^	-	-	34.6 (±5.7) ^a^
	7 days	39.0 (±12.3) ^a^	32.7 (±5.2) ^a^	34.6 (±10.9) ^a^	30.9 (±16.0) ^a^
	14 days	43.6 (±18.4) ^a^	32.0 (±7.5) ^a^	35.8 (±7.0) ^a^	37.5 (±4.7) ^a^
	21 days	35.3 (±12.0) ^a^	22.0 (±7.0) ^a^	40.0 (±12.9) ^a^	34.7 (±21.4) ^a^
HDL-cholesterol (mg/dL)	baseline	38.4 (±13.5) ^a^	-	-	21.1 (±5.7) ^b^
	7 days	31.4 (±11.8) ^a^	32.0 (±5.7) ^a^	22.6 (±6.4) ^a^	19.7 (±8.3) ^a^
	14 days	35.8 (±14.7) ^a^	29.3 (±5.1) ^a,b^	24.4 (±7.1) ^a,b^	21.8 (±6.5) ^b^
	21 days	31.7 (±12.5) ^a^	30.7 (±9.6) ^a^	24.3 (±7.1) ^a^	20.0 (±8.2) ^a^
Total cholesterol (mg/dL)	baseline	87.0 (±40.6) ^a^	-	-	39.8 (±11.4) ^b^
	7 days	61.6 (±25.2) ^a^	61.1 (±10.2) ^a^	43.6 (±12.0) ^a,b^	35.1 (±15.3) ^b^
	14 days	65.7 (±28.0) ^a^	59.7 (±6.4) ^a,b^	47.0 (±12.1) ^a,b^	41.1 (±8.5) ^b^
	21 days	61.7 (±21.4) ^a^	59.2 (±22.9) ^a^	43.0 (±12.8) ^a^	37.0 (±17.4) ^a^
**Marker of insulin resistance**					
HOMA-IR	baseline	1.4 (±0.6) ^a^	-	-	0.5 (±0.2) ^b^
	7 days	1.2 (±0.5) ^a^	1.6 (±0.5) ^a^	0.5 (±0.2) ^b^	0.4(±0.2) ^b^
	14 days	1.7 (±0.7) ^a^	1.4 (±0.6) ^a^	0.7 (±0.2) ^b^	0.6 (±0.2) ^b^
	21 days	1.5 (±0.8) ^a^	1.6 (±0.4) ^a^	0.6 (±0.2) ^b^	0.4 (±0.2) ^b^

Values are expressed as mean (±standard deviation). ^a,b^ Different letters indicate statistical differences among groups.

**Table 2:** Composition of the high-fat and control diets.

	Diet	
Ingredients (g/100 g)	High-Fat	Control
Corn starch	14.95	42.75
Casein	20.00	20.00
Dextrinized corn starch	10.00	13.20
Sucrose	10.00	10.00
Soybean oil	4.00	4.00
Cellulose	5.00	5.00
Mineral mix (AIN 93G) *	3.50	3.50
Vitamin mix (AIN 93) *	1.00	1.00
L-cystine	0.30	0.30
Choline bitartrate	0.25	0.25
Lard	31.00	-
Total	100.00	100.00
Energy (kcal/100 g)	540	385

* Composition following the recommendations of Reeves et al. [56].

## Data Availability

The data presented in this study are available on request from the corresponding author. The data are not publicly available due to ongoing related research.

## References

[1] World Health Organization (2022). Global Oral Health Status Report: Towards Universal Health Coverage for Oral Health by 2030.

[2] Chen M.X., Zhong Y.J., Dong Q.Q., Wong H.M., Wen Y.F. (2021). Global, regional, and national burden of severe periodontitis, 1990–2019: An analysis of the Global Burden of Disease Study 2019. J. Clin. Periodontol..

[3] Van Dyke T.E., Bartold P.M., Reynolds E.C. (2020). The Nexus Between Periodontal Inflammation and Dysbiosis. Front. Immunol..

[4] Trindade D., Carvalho R., Machado V., Chambrone L., Mendes J.J., Botelho J. (2023). Prevalence of periodontitis in dentate people between 2011 and 2020: A systematic review and meta-analysis of epidemiological studies. J. Clin. Periodontol..

[5] Albandar J.M., Susin C., Hughes F.J. (2018). Manifestations of systemic diseases and conditions that affect the periodontal attachment apparatus: Case definitions and diagnostic considerations. J. Periodontol..

[6] Papapanou P.N., Sanz M., Buduneli N., Dietrich T., Feres M., Fine D.H., Flemmig T.F., Garcia R., Giannobile W.V., Graziani F. (2018). Periodontitis: Consensus report of workgroup 2 of the 2017 World Workshop on the Classification of Periodontal and Peri-Implant Diseases and Conditions. J. Clin. Periodontol..

[7] American Diabetes Association Professional Practice Committee (2024). 2. Diagnosis and Classification of Diabetes: Standards of Care in Diabetes-2024. Diabetes Care.

[8] Atlas D. (2017). IDF Diabetes Atlas.

[9] Ahmad E., Lim S., Lamptey R., Webb D.R., Davies M.J. (2022). Type 2 diabetes. Lancet.

[10] American Diabetes Association Professional Practice Committee (2024). 8. Obesity and Weight Management for the Prevention and Treatment of Type 2 Diabetes: Standards of Care in Diabetes-2024. Diabetes Care.

[11] Ruze R., Liu T., Zou X., Song J., Chen Y., Xu R., Yin X., Xu Q. (2023). Obesity and type 2 diabetes mellitus: Connections in epidemiology, pathogenesis, and treatments. Front. Endocrinol..

[12] Graves D.T., Ding Z., Yang Y. (2020). The impact of diabetes on periodontal diseases. Periodontol. 2000.

[13] Su Y., Ye L., Hu C., Zhang Y., Liu J., Shao L. (2023). Periodontitis as a promoting factor of T2D: Current evidence and mechanisms. Int. J. Oral Sci..

[14] Stohr J., Barbaresko J., Neuenschwander M., Schlesinger S. (2021). Bidirectional association between periodontal disease and diabetes mellitus: A systematic review and meta-analysis of cohort studies. Sci. Rep..

[15] Hajishengallis G., Chavakis T. (2021). Local and systemic mechanisms linking periodontal disease and inflammatory comorbidities. Nat. Rev. Immunol..

[16] Graves D.T., Kang J., Andriankaja O., Wada K., Rossa C. (2012). Animal models to study host-bacteria interactions involved in periodontitis. Front. Oral Biol..

[17] Bodet C., Chandad F., Grenier D. (2007). Pathogenic potential of *Porphyromonas gingivalis*, *Treponema denticola* and *Tannerella forsythia*, the red bacterial complex associated with periodontitis. Pathol. Biol..

[18] Acqua Y.D., Hernandez C., Fogacci M., Barbirato D., Palioto D. (2022). Local and systemic effects produced in different models of experimental periodontitis in mice: A systematic review. Arch. Oral Biol..

[19] Mysak J., Podzimek S., Sommerova P., Lyuya-Mi Y., Bartova J., Janatova T., Prochazkova J., Duskova J. (2014). *Porphyromonas gingivalis*: Major periodontopathic pathogen overview. J. Immunol. Res..

[20] Liu F., Zhu B., An Y., Zhou Z., Xiong P., Li X., Mi Y., He T., Chen F., Wu B. (2024). Gingipain from Porphyromonas gingivalis causes insulin resistance by degrading insulin receptors through direct proteolytic effects. Int. J. Oral Sci..

[21] Furman B.L. (2021). Streptozotocin-Induced Diabetic Models in Mice and Rats. Curr. Protoc..

[22] Wang B., Chandrasekera P.C., Pippin J.J. (2014). Leptin- and leptin receptor-deficient rodent models: Relevance for human type 2 diabetes. Curr. Diabetes Rev..

[23] King A.J. (2012). The use of animal models in diabetes research. Br. J. Pharmacol..

[24] Skovso S. (2014). Modeling type 2 diabetes in rats using high fat diet and streptozotocin. J. Diabetes Investig..

[25] de Molon R.S., de Avila E.D., Boas Nogueira A.V., Chaves de Souza J.A., Avila-Campos M.J., de Andrade C.R., Cirelli J.A. (2014). Evaluation of the host response in various models of induced periodontal disease in mice. J. Periodontol..

[26] de Molon R.S., Mascarenhas V.I., de Avila E.D., Finoti L.S., Toffoli G.B., Spolidorio D.M., Scarel-Caminaga R.M., Tetradis S., Cirelli J.A. (2016). Long-term evaluation of oral gavage with periodontopathogens or ligature induction of experimental periodontal disease in mice. Clin. Oral Investig..

[27] Lou J., Zhang B., Cai J., Zhang L., Zhao Y., Zhao Z. (2025). Diabetes exacerbates periodontitis by disrupting IL-33-mediated interaction between periodontal ligament fibroblasts and macrophages. Int. Immunopharmacol..

[28] Fu X., Liu B., Sun J., Zhang X., Zhu Z., Wang H., Xiao A., Gan X. (2023). Perturbation of mitochondrial dynamics links to the aggravation of periodontitis by diabetes. J. Histotechnol..

[29] Cavagni J., de Macedo I.C., Gaio E.J., Souza A., de Molon R.S., Cirelli J.A., Hoefel A.L., Kucharski L.C., Torres I.L., Rosing C.K. (2016). Obesity and Hyperlipidemia Modulate Alveolar Bone Loss in Wistar Rats. J. Periodontol..

[30] Ranbhise J.S., Ju S., Singh M.K., Han S., Akter S., Ha J., Choe W., Kim S.S., Kang I. (2025). Chronic Inflammation and Glycemic Control: Exploring the Bidirectional Link Between Periodontitis and Diabetes. Dent. J..

[31] Chen X., He Y., Zhou Y., Gong H., Zhang J., Qiu G., Shen Y., Qin W. (2025). Modulatory role of exogenous arachidonic acid in periodontitis with type 2 diabetes mellitus mice. BMC Oral Health.

[32] Li Y., Lu Z., Zhang L., Kirkwood C.L., Kirkwood K.L., Lopes-Virella M.F., Huang Y. (2022). Inhibition of acid sphingomyelinase by imipramine abolishes the synergy between metabolic syndrome and periodontitis on alveolar bone loss. J. Periodontal Res..

[33] Li L., Qin W., Ye T., Wang C., Qin Z., Ma Y., Mu Z., Jiao K., Tay F.R., Niu W. (2025). Bioactive Zn-V-Si-Ca Glass Nanoparticle Hydrogel Microneedles with Antimicrobial and Antioxidant Properties for Bone Regeneration in Diabetic Periodontitis. ACS Nano.

[34] Fang X., Wang J., Ye C., Lin J., Ran J., Jia Z., Gong J., Zhang Y., Xiang J., Lu X. (2024). Polyphenol-mediated redox-active hydrogel with H_2_S gaseous-bioelectric coupling for periodontal bone healing in diabetes. Nat. Commun..

[35] Gu Y., Golub L.M., Lee H.M., Walker S.G. (2025). Diabetes, periodontal disease, and novel therapeutic approaches- host modulation therapy. Front. Clin. Diabetes Healthc..

[36] Heydemann A. (2016). An Overview of Murine High Fat Diet as a Model for Type 2 Diabetes Mellitus. J. Diabetes Res..

[37] Racine K.C., Iglesias-Carres L., Herring J.A., Wieland K.L., Ellsworth P.N., Tessem J.S., Ferruzzi M.G., Kay C.D., Neilson A.P. (2024). The high-fat diet and low-dose streptozotocin type-2 diabetes model induces hyperinsulinemia and insulin resistance in male but not female C57BL/6J mice. Nutr. Res..

[38] Winzell M.S., Ahren B. (2004). The high-fat diet-fed mouse: A model for studying mechanisms and treatment of impaired glucose tolerance and type 2 diabetes. Diabetes.

[39] Costa M.C., Lima T.F.O., Arcaro C.A., Inacio M.D., Batista-Duharte A., Carlos I.Z., Spolidorio L.C., Assis R.P., Brunetti I.L., Baviera A.M. (2020). Trigonelline and curcumin alone, but not in combination, counteract oxidative stress and inflammation and increase glycation product detoxification in the liver and kidney of mice with high-fat diet-induced obesity. J. Nutr. Biochem..

[40] Wang Q., Zhang P., Aprecio R., Zhang D., Li H., Ji N., Mohamed O., Zhang W., Li Y., Ding Y. (2016). Comparison of Experimental Diabetic Periodontitis Induced by *Porphyromonas gingivalis* in Mice. J. Diabetes Res..

[41] Huang K.C., Chuang P.Y., Yang T.Y., Tsai Y.H., Li Y.Y., Chang S.F. (2024). Diabetic Rats Induced Using a High-Fat Diet and Low-Dose Streptozotocin Treatment Exhibit Gut Microbiota Dysbiosis and Osteoporotic Bone Pathologies. Nutrients.

[42] Xepapadaki E., Nikdima I., Sagiadinou E.C., Zvintzou E., Kypreos K.E. (2021). HDL and type 2 diabetes: The chicken or the egg?. Diabetologia.

[43] Hahn M., van Krieken P.P., Nord C., Alanentalo T., Morini F., Xiong Y., Eriksson M., Mayer J., Kostromina E., Ruas J.L. (2020). Topologically selective islet vulnerability and self-sustained downregulation of markers for beta-cell maturity in streptozotocin-induced diabetes. Commun. Biol..

[44] Li P., Fan C., Cai Y., Fang S., Zeng Y., Zhang Y., Lin X., Zhang H., Xue Y., Guan M. (2020). Transplantation of brown adipose tissue up-regulates miR-99a to ameliorate liver metabolic disorders in diabetic mice by targeting NOX4. Adipocyte.

[45] Chao P.C., Li Y., Chang C.H., Shieh J.P., Cheng J.T., Cheng K.C. (2018). Investigation of insulin resistance in the popularly used four rat models of type-2 diabetes. Biomed. Pharmacother..

[46] Martinez-Garcia M., Hernandez-Lemus E. (2021). Periodontal Inflammation and Systemic Diseases: An Overview. Front. Physiol..

[47] Palioto D.B., Finoti L.S., Kinane D.F., Benakanakere M. (2019). Epigenetic and inflammatory events in experimental periodontitis following systemic microbial challenge. J. Clin. Periodontol..

[48] Murugaiyan V., Utreja S., Hovey K.M., Sun Y., LaMonte M.J., Wactawski-Wende J., Diaz P.I., Buck M.J. (2024). Defining *Porphyromonas gingivalis* strains associated with periodontal disease. Sci. Rep..

[49] Haugsten H.R., Kristoffersen A.K., Haug T.M., Soland T.M., Ovstebo R., Aass H.C.D., Enersen M., Galtung H.K. (2023). Isolation, characterization, and fibroblast uptake of bacterial extracellular vesicles from *Porphyromonas gingivalis* strains. Microbiologyopen.

[50] Blasco-Baque V., Garidou L., Pomie C., Escoula Q., Loubieres P., Le Gall-David S., Lemaitre M., Nicolas S., Klopp P., Waget A. (2017). Periodontitis induced by *Porphyromonas gingivalis* drives periodontal microbiota dysbiosis and insulin resistance via an impaired adaptive immune response. Gut.

[51] Tian J., Liu C., Zheng X., Jia X., Peng X., Yang R., Zhou X., Xu X. (2020). *Porphyromonas gingivalis* Induces Insulin Resistance by Increasing BCAA Levels in Mice. J. Dent. Res..

[52] Cai Z., Du S., Zhao N., Huang N., Yang K., Qi L. (2024). Periodontitis promotes the progression of diabetes mellitus by enhancing autophagy. Heliyon.

[53] Zhang B., Yang Y., Yi J., Zhao Z., Ye R. (2021). Hyperglycemia modulates M1/M2 macrophage polarization via reactive oxygen species overproduction in ligature-induced periodontitis. J. Periodontal Res..

[54] Xiao E., Mattos M., Vieira G.H.A., Chen S., Correa J.D., Wu Y., Albiero M.L., Bittinger K., Graves D.T. (2017). Diabetes Enhances IL-17 Expression and Alters the Oral Microbiome to Increase Its Pathogenicity. Cell Host Microbe.

[55] Canadian Council on Animal Care (1993). Guide to the Care and Use of Experimental Animals.

[56] Reeves P.G., Nielsen F.H., Fahey G.C. (1993). AIN-93 purified diets for laboratory rodents: Final report of the American Institute of Nutrition ad hoc writing committee on the reformulation of the AIN-76A rodent diet. J. Nutr..

[57] De Avila E.D., Castro A.G., Tagit O., Krom B.P., Löwik D., Van Well A.A., Bannenberg L.J., Vergani C.E., Van den Beucken J.J. (2019). Anti-bacterial efficacy via drug-delivery system from layer-by-layer coating for percutaneous dental implant components. Appl. Surf. Sci..

[58] Wang Y., Yu X., Lin J., Hu Y., Zhao Q., Kawai T., Taubman M.A., Han X. (2017). B10 Cells Alleviate Periodontal Bone Loss in Experimental Periodontitis. Infect. Immun..

[59] Lee S., Muniyappa R., Yan X., Chen H., Yue L.Q., Hong E.G., Kim J.K., Quon M.J. (2008). Comparison between surrogate indexes of insulin sensitivity and resistance and hyperinsulinemic euglycemic clamp estimates in mice. Am. J. Physiol. Endocrinol. Metab..

[60] Nogueira A.V., de Molon R.S., Nokhbehsaim M., Deschner J., Cirelli J.A. (2017). Contribution of biomechanical forces to inflammation-induced bone resorption. J. Clin. Periodontol..

[61] Longhini R., Aparecida de Oliveira P., Sasso-Cerri E., Cerri P.S. (2014). Cimetidine reduces alveolar bone loss in induced periodontitis in rat molars. J. Periodontol..

[62] Silva R.C.L., Sasso-Cerri E., Cerri P.S. (2022). Diacerein-induced interleukin-1beta deficiency reduces the inflammatory infiltrate and immunoexpression of matrix metalloproteinase-8 in periodontitis in rat molars. J. Periodontol..

[63] Fernandes N.A.R., Camilli A.C., Maldonado L.A.G., Pacheco C.G.P., Silva A.F., Molon R.S., Spolidorio L.C., Ribeiro de Assis L., Regasini L.O., Rossa Junior C. (2021). Chalcone T4, a novel chalconic compound, inhibits inflammatory bone resorption in vivo and suppresses osteoclastogenesis in vitro. J. Periodontal Res..

[64] Souza J.A.C., Magalhaes F.A.C., Oliveira G., Molon R.S., Zuanon J.A., Souza P.P.C. (2020). Pam2CSK4 (TLR2 agonist) induces periodontal destruction in mice. Braz. Oral Res..

